# Modulating the sialic acid content of endothelial cells affects their adhesion and barrier permeability

**DOI:** 10.1038/s41598-026-66496-z

**Published:** 2026-08-19

**Authors:** Paula M. Müller, Linus Weilepp, Kaya Bork, Rüdiger Horstkorte, Astrid Gesper

**Affiliations:** Institute for Physiological Chemistry, Medical Faculty, Martin-Luther-University Halle-Wittenberg, 06114 Halle (Saale), Germany

**Keywords:** Glycobiology, Podocalyxin, Integrin beta-1, Endothelial cells, Sialylation, UDP-*N*-acetylglucosamine 2-epimerase/*N*-acetylmannosamine kinase, Biochemistry, Cell biology

## Abstract

The crossing of the endothelial cell layer and the glycocalyx that shields it can be considered the final barrier for substances trying to pass from the blood into the surrounding tissue. Due to their terminal position on glycan structures, sialic acids constitute the initial point of contact encountered by invading substances or bacteria. In this study, we investigated the influence of varying sialic acid levels on endothelial cell adhesion and barrier permeability. Furthermore, the successful desialylation as well as the potential for resialylation of two natively sialylated proteins – podocalyxin and integrin beta-1 – were investigated in detail. Desialylation of the cells was achieved either by sialidase-treatment or by knocking out the key enzyme of the sialic acid biosynthesis – the UDP-*N*-acetylglucosamine 2-epimerase/*N*-acetylmannosamine kinase (GNE). We could confirm a sialic acid deficiency after both approaches, which was more pronounced in the *GNE*-KO cells than in the sialidase-treated cells. Furthermore, a reduced barrier permeability and a negative effect on adhesion were observed. Partial resialylation of *GNE*-KO cells was achieved either by supplementation with *N-*acetylmannosamine (ManNAc) or with *N-*acetylneuraminic acid (Neu5Ac); however, the effects on adhesion and permeability were not fully compensated thereby.

**Supplementary Information:**

The online version contains supplementary material available at 10.1038/s41598-026-66496-z.

## Introduction

The degree of protein sialylation has a significant influence on the half-life of proteins^1,2^ and, in the case of plasma proteins, also affects the hepatic clearance rate^3^. Furthermore, it can affect cell migration^4^, integrin-mediated adhesion to ligands and substrates^4–6^, the metastasis of tumor cells^7,8^, as well as the function of ion channels (see e.g. review^9^).

**Fig. 1:**
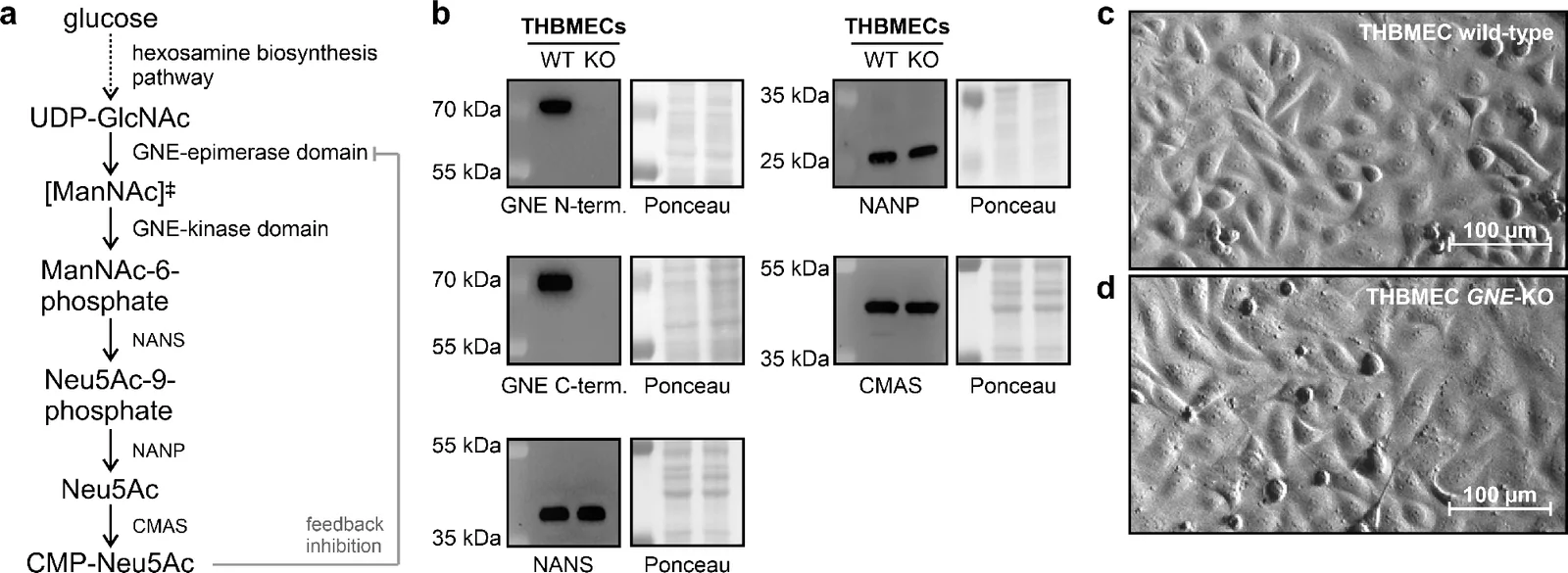
Western blots of enzymes of the sialic acid biosynthesis pathway and microscopy images of wild-type and *GNE*-knockout THBMECs. Scheme of the sialic acid biosynthesis pathway (**a**). First, glucose is metabolized to UDP-GlcNAc via the hexosamine biosynthesis pathway (HBP). Subsequently, UDP-GlcNAc is epimerized and phosphorylated by the two domains of the GNE resulting in the formation of ManNAc-6-phosphate. Afterwards, Neu5Ac-9-phosphate is synthesized by the *N*-acetylneuraminate-9-phosphate synthase (NANS). This is then dephosphorylated by the *N*-acylneuraminate-9-phosphatase (NANP) and activated by *N*-acylneuraminate cytidyltransferase (CMAS) with CMP, resulting in CMP-Neu5Ac, which can then serve as a substrate for sialyltransferases and can also feedback inhibit the GNE-epimerase domain. To investigate the effect of the *GNE*-knockout on the expression levels of the other enzymes in the sialic acid biosynthesis pathway, we analyzed cell lysates by Western blot (**b**). Representative phase contrast images of both THBMEC cell lines – wild-type (**c**) and *GNE*-KO (**d**). For reasons of space, only the abbreviation “KO” for “*GNE*-KO” is used in the labeling of the Western blots. All Western blots resulted from three independent cell culture lyses that were investigated individually (n = 3). Furthermore, all Western blots were cropped. The original blots can be found in the Supplementary Dataset File. Information concerning the antibodies used and their concentration can be found in Table 1 in the Methods section. The phase contrast images were acquired using an Axiovert 100 (ZEISS, Germany) with 5x magnification and the AxioCam ICc 1 (ZEISS, Germany).

In humans, the sialic acid *N*-acetylneuraminic acid (Neu5Ac) predominates almost exclusively^10,11^. The synthesis of Neu5Ac begins with UDP-*N*-acetylglucosamine (UDP-GlcNAc), which has been formed upstream via the hexosamine biosynthesis pathway (HBP), starting from glucose (scheme can be seen in Fig. 1a). UDP-GlcNAc is first epimerized towards *N*-acetylmannosamine (ManNAc) by the epimerase- and subsequently phosphorylated to ManNAc-6-phosphate by the kinase-domain of the UDP-*N*-acetylglucosamine 2-epimerase/*N*-acetylmannosamine kinase (GNE; epimerase-domain EC 3.2.1.183 and kinase-domain EC 2.7.1.60). Afterwards, the *N*-acetylneuraminate-9-phosphate synthase (NANS; EC 2.5.1.57) further metabolized it to Neu5Ac-9-phosphate, which is then dephosphorylated by the *N-*acylneuraminate-9-phosphatase (NANP; EC 3.1.3.29). The resulting sialic acid is subsequently activated to CMP-Neu5Ac by the *N-*acylneuraminate cytidyltransferase (CMAS; EC 2.7.7.43) and can then serve as a substrate for sialyltransferases.

In this study, we induced global desialylation in cultured transfected human microvascular endothelial cells from the brain (THBMECs;^12^) and investigated its effects on adhesion and barrier integrity, as these cells are known to be suitable for establishing a highly simplified model of the blood-brain barrier^13,14^. Desialylation was achieved using two different methods: one aimed at directly influencing the pathway, while the other involved the enzymatic removal of sialic acids from the completed glycan structures. For the first method, we decided to knock-out GNE, and for the second method, the cells were treated with sialidase from *Arthrobacter ureafaciens.* As read-out parameter for desialylation, we specifically investigated the natively sialylated proteins podocalyxin and integrin beta-1^15,16^- in addition to determining changes in total sialic acid content. Furthermore, it is known that barrier integrity can be influenced by podocalyxin expression^17^, and adhesion by the sialylation status of e.g. integrin beta-1^4,5^. Thus, the choice of these proteins is meaningful not only as a read-out parameter for desialylation.

We were able to show that the desialylation of podocalyxin reduced its electrophoretic mobility, whereas that of integrin beta-1 was increased. Furthermore, the desialylation effect was more pronounced in the *GNE*-knockout cells than in the sialidase-treated cells. In the knockout cells, this could be partially compensated for by supplementation with ManNAc or Neu5Ac. Additionally, desialylation of the cells reduced their adhesion to a fibronectin-collagen IV substrate and resulted in the formation of a tighter barrier. Following the discontinuation of sialidase-treatment, normosialylated podocalyxin was detectable again in the Western blot at the 6-hour time point; for integrin beta-1, this was not the case until the 9-hour time point. In both cases, the reappearance of the normosialylated protein was presumably attributable to *de novo* synthesis. In summary, this study contributes to a better understanding of the effects of global desialylation on endothelial cells.

## Results and discussion

### Generation of a human endothelial GNE-knockout cell line

In the first part of this study, we generated a human endothelial cell line (THBMEC;^12^) with a knockout of UDP-*N*-acetylglucosamine 2-epimerase/*N*-acetylmannosamine kinase (GNE), one of the key enzymes in the sialic acid biosynthesis pathway (Fig. 1a). Afterwards, the protein expression levels of the remaining enzymes in this metabolic pathway were examined, since it seems plausible that, if the key enzyme is no longer present, the other enzymes are subsequently downregulated.

All enzymes of the sialic acid biosynthesis pathway were expressed in both THBMEC cell lines (Fig. 1b; all raw blot images could be found in the Supplementary Dataset file on the corresponding tab), with no significant expression differences observed; except for GNE, which was absent in the knockout cells but therefore perfectly matched the expected phenotype. Furthermore, we examine the cell morphology using microscopic images of both cell lines and observed no differences (Fig. 1c (wild-type) and d (*GNE*-knockout)).

From this, it can be concluded that the knockout was successful and the expression levels of the other enzymes in the sialic acid biosynthesis pathway were not directly affected.

### Determination of the sialylation status of GNE-knockout and sialidase-treated wild-type cells and their impact on barrier permeability and adhesion

**Fig. 2:**
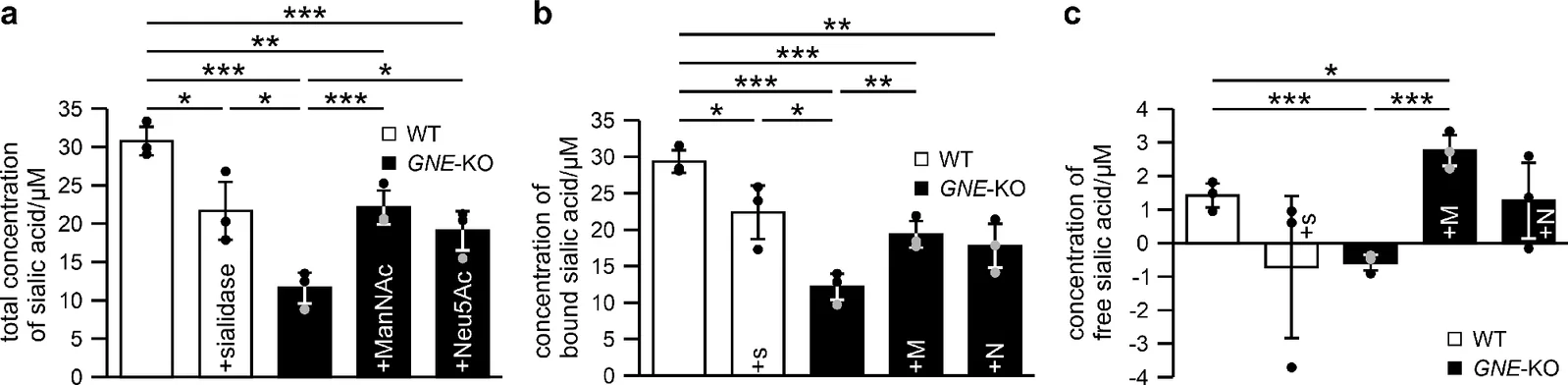
Determination of the sialic acid content of wild-type, sialidase-treated, *GNE*-knockout and supplemented *GNE*-knockout THBMECs by the periodate-resorcinol assay. (**a**) shows in a bar chart the total concentration of sialic acid, (**b**) the concentration of glycoconjugate-bound sialic acid, and (**c**) the concentration of free sialic acid of the above-mentioned cells. The abbreviation “+s” in panels (**b**) and (**c**) indicates the addition of sialidase, “+M” of ManNAc, and “+N” of Neu5Ac. Sialidase was used at a concentration of 10 mU/mL, ManNAc at 5 mM, and Neu5Ac likewise at 5 mM. All experiments resulted from three independent cell culture lyses that were investigated simultaneously (*n* = 3). The height of the bar indicates the mean and the error bar indicates the standard deviation. Significance was determined using a two-sample *t*-test assuming different variances (one-sided). 1.00E-02 < *p* ≤ 5.00E-02: *, 5.00E-03 < *p* ≤ 1.00E-02: **, and *p* ≤ 5.00E-03: *** Exact *p*-values and raw data are available in the Supplementary Dataset File.

Next, we aimed to investigate the effects of the *GNE*-knockout on sialic acid content, adhesion, and barrier integrity. As a second approach to modulating the sialylation of a cell, we treated wild-type cells with sialidase and performed the same experiments with them as with the *GNE*-knockout cells. The sialic acid content was determined using the periodate-resorcinol assay. We found evidence of hyposialylation in both cell lines with total sialic acid reduced by 30% in sialidase-treated cells (*p-*value 2.76E-02) and by 62% in *GNE*-knockout cells (*p-*value 2.94E-04). The same pattern was observable for glycoconjugate-bound sialic acid, the content of which was reduced by 24% in sialidase-treated cells (*p*-value 4.51E-02) and by 58% in *GNE*-knockout cells (*p-*value 2.59E-04) (Fig. 2a and b). Hyposialylation in the *GNE*-knockout cells could be partially reversed by treatment with ManNAc or Neu5Ac, although the values still differed significantly from those found in the wild-type cells. All reduction values given here refer to the comparison to the wild-type value and are based on a comparison of the mean values. However, the decrease in sialic acid levels in the sialidase-treated cells was not as pronounced as in the *GNE*-knockout cells. This is consistent with the fact that GNE is a very important enzyme in the sialic acid biosynthesis pathway (see e.g.^18^); accordingly, a deficiency of this enzyme should have more far-reaching effects than treatment with sialidase, which also does not have the same affinity for all possible sialic acid binding types.

Furthermore, calculating the concentration of free, unbound sialic acid, which is simply the total concentration minus the concentration of sialic acid bound to glycoconjugates, revealed that we found almost no free sialic acid in *GNE*-knockout cells (*p*-value 3.46E-03; Fig. 2c). We concluded that our *GNE*-knockout cells are in an absolute sialic acid deficiency situation, where any free sialic acid would be immediately metabolized and added to glycoconjugates to maintain basic cell functions for as long as possible. The fact that we were still able to detect a level of 38% of the total sialic acid level of the untreated wild-type cells in the *GNE*-knockout cells is in line with the results of Peters et al., where some residual sialic acid content was also detected in HEK-293 *GNE*-knockout cells^19^. This could be due to a basal content of sialic acids in the FCS-containing medium used^20^ or to alternative branches of the sialic acid biosynthesis pathway, which, although apparently unable to prevent hyposialylation, could still provide a minimal supply to ensure cell survival. However, the exact nature of these branches still needs to be further investigated.

**Fig. 3:**
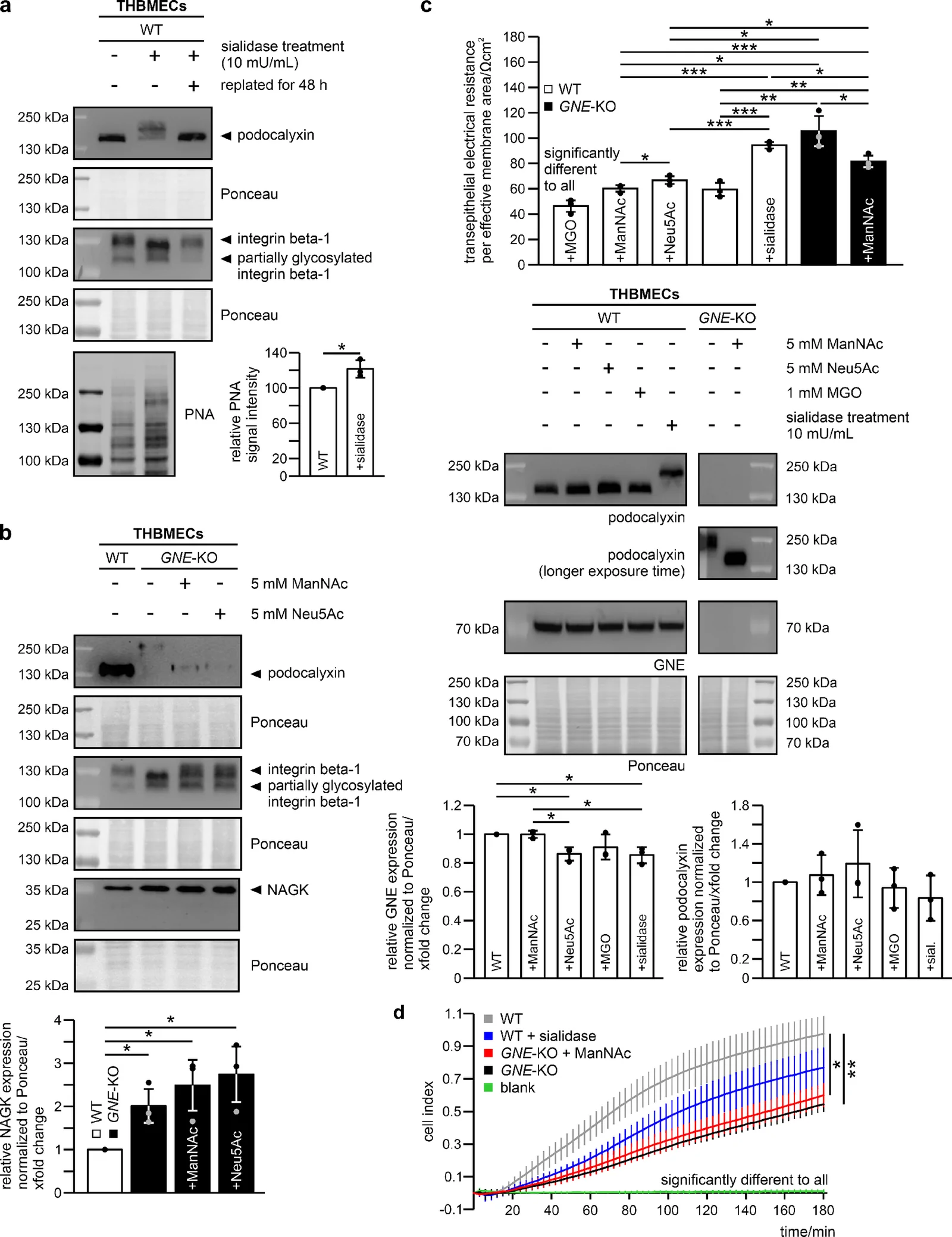
Detection of desialylation using two natively sialylated marker proteins, podocalyxin and integrin beta-1, in Western blot and investigation of the effects of global desialylation on barrier permeability and adhesion. Representative Western Blots of podocalyxin, integrin beta-1, and AlexaFluor647-labeled *Arachis* *hypogaea* lectin (PNA) from cell lysates of untreated cells, sialidase-treated and sialidase-treated and replated cells (**a**). Representative Western Blot of podocalyxin, integrin beta-1, and NAGK from untreated wild-type, untreated *GNE*-knockout cells, *GNE*-knockout cells treated with 5 mM ManNAc, and *GNE*-knockout cells treated with 5 mM Neu5Ac (**b**). Results on barrier permeability by determining the transepithelial electrical resistance (TEER) (**c**, upper part) and the corresponding Western blots for podocalyxin and GNE, the samples of which were obtained directly from the TEER-insets (**c**, lower part). Results on the adhesion (**d**) of differently treated cells to a fibronectin-collagen IV substrate. All Western blot experiments, barrier permeability and adhesion measurements were performed three times (n = 3). The samples for the PNA-analysis were analyzed together on a single blot, but they originate from three independent sample generation processes. For TEER and RTCA measurements: For each approach, three technical replicates were prepared and averaged – giving the value for one biological replicate. The order of the conditions in the legend in (**d**) represents the order of the graphs at the end of the measurement – meaning that WT is the top graph and blank the lowest. The height of the bars (**a, b, c**) or the measurement points in (**d**) indicates the mean and the error bar indicates the standard deviation. Significance was determined using a two-sample *t*-test assuming different variances (one-sided). 1.00E-02 <* p* ≤ 5.00E-02: *, 5.00E-03 < *p* ≤ 1.00E-02: **, and *p* ≤ 5.00E-03: *** Exact *p*-values and raw data are available in the Supplementary Dataset File. Furthermore, all Western blots were cropped. During the analysis of the Western blot showing the PNA-lectin staining, contrast, brightness, and intensity were adjusted - with the processing applied to the entire blot to ensure consistent adjustments throughout. The original blot can also be found in the Supplementary Dataset File. Information concerning the antibodies used and their concentration can be found in Table 1 in the Methods section.

As a second indicator of the sialylation status of a cell, we analyzed the sialic acid carrier proteins podocalyxin and integrin beta-1 after sialidase-treatment in Western blot experiments (Fig. 3a – upper and middle part). Both podocalyxin and integrin beta-1 are transmembrane proteins^21,22^; furthermore, podocalyxin belongs to the family of mucin domain-containing proteins^21^, whereas integrin beta-1 plays a crucial role in adhesion processes^4,5^. It was found that sialidase-treated podocalyxin exhibited lower electrophoretic mobility compared to untreated podocalyxin and therefore appeared as a band at a higher molecular weight in the Western blot. This phenomenon has already been demonstrated by other research groups^23,24^ and contradicts the notion that molecular weight must decrease upon the removal of a component – a premise we were nevertheless able to successfully demonstrate using integrin beta-1 as an example (Fig. 3a – middle part).

The untreated integrin beta-1 appeared as two bands in the Western blot; one band was found at approximately 130 kDa and the other at about 110 kDa. The lower molecular weight band represents the partially glycosylated ER form of integrin beta-1^25^. Whereas the other band represents the fully glycosylated mature form of integrin beta-1. Following sialidase-treatment, the upper band shifted downwards, indicating higher electrophoretic mobility, whereas the lower band remained unchanged. This is consistent with the fact that this form of integrin beta-1 is not yet sialylated.

The behavior of podocalyxin during gel electrophoresis can be explained by the theory of Gahmberg and Andersson, who hypothesized that the charge of sialic acid – under certain conditions or in specific structural contexts – can influence a protein’s electrophoretic mobility^26^. Furthermore, glycans can shield the protein backbone from SDS, allowing the protein’s charge to directly influence its electrophoretic mobility. In addition to that, podocalyxin is sulfated, which likewise introduces negative charge^23^. The change in the electrophoretic mobility was reversible for both proteins 48 h after replating following sialidase-treatment.

Subsequently, we investigated the presence of Galβ1-3GalNAcα1-O-Ser/Thr using *Arachis hypogaea* lectin (PNA)^27^ in Western Blot analysis. We observed an increase by 22% in total signal intensity following sialidase-treatment (*p*-value 3.18E-02; Fig. 3a – lower part); this indicates, that, under normal conditions, many of these structures are masked from the lectin by Neu5Ac and become accessible after sialidase-treatment. This can be regarded as an indirect indication of hyposialylation following treatment with sialidase.

In the next step, we investigated the electrophoretic mobility of podocalyxin and integrin beta-1 in *GNE*-knockout cells and observed the same changes in electrophoretic mobility that had previously been seen in sialidase-treated cells (Fig. 3b - upper and middle part). Treatment with ManNAc or Neu5Ac reversed this change. The possibility of restoring the sialylation status of podocalyxin by ManNAc supplementation has already been demonstrated in mice that carried mutations in the *Gne* gene and therefore also had hyposialylated podocalyxin^28,29^. Nevertheless, the question remains as to how the *GNE*-knockout cells and the *Gne*-variant mice can metabolize ManNAc. Of course, in principle, the Gne is still present in the mice and shows some residual activity, but another study has already shown that supplemented ManNAc is unlikely to be metabolized by the GNE^30^.

There are several hypotheses as to how it can actually be metabolized^19^– the question of whether this is possible in the cells studied has already been confirmed by this experiment. One hypothesis, for example, states that the phosphorylation step can alternatively be carried out by GlcNAc kinase (NAGK; EC 2.7.1.60;^31,32^). Therefore, we next examined the expression of the NAGK and found that it is 2fold increased in the untreated (*p*-value 3.35E-02), 2.5fold in the ManNAc- (*p*-value 3.50E-02), and 2.7fold in the Neu5Ac-treated *GNE*-knockout cells (*p*-value 3.08E-02) compared to the wild-type (Fig. 3b - lower part). This could be seen as an indication of a possible compensatory role of NAGK; then, at least supplemented ManNAc could be converted into new sialic acid. Furthermore, it is interesting that supplementation of Neu5Ac also increased NAGK expression, as one might simply assume that ingested Neu5Ac could be directly activated by CMAS. However, this result suggests a different metabolism of the presumably endocytotically taken up Neu5Ac.

Furthermore, it is noticeable that the signal intensities for podocalyxin in the *GNE*-knockout cell lysates - whether untreated or treated - appeared a lot weaker than in the untreated wild-type sample. Decreased signal intensities typically indicates lower protein expression. Nevertheless, since it is not known whether antibody affinity to normosialylated and hyposialylated podocalyxin is the same, such a statement must be made with caution. However, since the band remains similarly weak after supplementation, it can be assumed that the expression of podocalyxin in the *GNE*-knockout cells might actually be reduced. This is certainly interesting, as no direct interaction between GNE and podocalyxin is known to date. The only known connection to date is that podocalyxin was found to be hyposialylated in *Gne*-mutant mouse models^28,29,33^. These models were characterized in particular by exhibiting a clear renal, but no muscular phenotype, which is especially interesting as patients carrying mutations in the *GNE* gene exhibit a very pronounced muscular phenotype with virtually no nephrological anomalies^34^. By analyzing the interaction partners of podocalyxin and GNE published on BioGRID (data retrieved on 17th June 2026^35^), we were able to identify six potential linking partners (HDAC2, RPN2, TRIM67, HEXB, and SUCLG2). Whether one of them truly functions as a linking partner, remains to be investigated in future studies.

Besides, differences in the position of the hyposialylated podocalyxin band are evident between the two sialylation modulation conditions. Podocalyxin from *GNE*-knockout cells showed an even lower electrophoretic mobility than that from sialidase-treated cells. This could again indicate that the degree of podocalyxin hyposialylation is higher in the *GNE*-knockout cells, as already suggested in the evaluation of the periodate-resorcinol assay.

Furthermore, we investigated the effect of hyposialylation on the transepithelial electrical resistance per effective membrane area as an indicator of barrier permeability (Fig. 3c – upper part). As negative control, the cells were treated with 1 mM methylglyoxal (MGO), which is known to have a negative effect on membrane permeability^36^. We were able to confirm this in our experiment and found a significantly lower resistance compared to all other conditions. Furthermore, we observed a 1.59fold increase in resistance after sialidase-treatment (*p-*value 1.67E-03) and a 1.78fold increase in the *GNE*-knockout cells (*p-*value 7.56E-03) compared to the untreated wild-type cells. Treating the *GNE*-knockout cells with ManNAc partly reversed the increase in resistance by -23% (compared to untreated *GNE*-knockout cells; *p-*value 3.82E-02), although resistance in these cells remained 1.37times higher than in untreated wild-type cells (*p-*value 5.13E-03). Treatment of the wild-type cells with ManNAc or Neu5Ac resulted in no significant changes compared to the untreated wild-type cells. Overall, we conclude that a reduced level of sialylation in THBMECs leads to lower membrane permeability and better barrier integrity, which is consistent with the data from the Takeda group on sialidase-treated Madine-Darby canine kidney (MDCK) cells^24^. A possible explanation could be that the negative charges, e.g. on podocalyxin, can lead to a mutual anti-adhesive behavior between two cells and that this can be reversed to an adhesive behavior by removing sialic acids. This would be consistent with the results of a podocalyxin knockout in mice, where slit diaphragms were no longer formed and instead the presence of tight junctions reduced permeability^37^.

Furthermore, we were able to harvest the cells after the TEER-measurements and subsequently examine the expression levels of podocalyxin and GNE (Fig. 3c – lower part). A GNE expression signal was detectable only in the wild-type cells, consistent with the expected phenotype. Further, we observed a 0.86fold decrease in GNE expression after the cells had been continuously supplied with Neu5Ac (*p*-value 1.86E-02) and a 0.94fold decrease after continuous treatment with sialidase (*p*-value 2.31E-02). This downregulation after Neu5Ac supplementation is consistent with the fact that its metabolism is independent of GNE and that its activated form, CMP-Neu5Ac, is further capable of inhibiting the GNE via feedback (see scheme in Fig. 1a). Interestingly, we did not found the same effect for ManNAc supplementation. Since it is hypothesized, that GNE cannot metabolize supplemented ManNAc^30^, its metabolism should occur independently of the GNE as well. Sialidase is an enzyme that is also expressed endogenously in cells, e.g. in the lysosome, for the cleavage of sialic acids from glycoconjugates^38^. Therefore, it is possible that their expression is also directly related to the production of sialic acid and thus to the expression of GNE, since an increased release of sialic acid – caused, for example, by increased sialidase expression – would under normal circumstances promise the free availability of Neu5Ac. Under these assumptions, it seems to make sense to downregulate GNE expression through continuous administration of sialidase.

For podocalyxin, we again observed a shift in electrophoretic mobility following sialidase-treatment as well as in untreated *GNE*-knockout cells. The expression levels in the wild-type cells showed no significant difference – even after sialidase-treatment. In contrast, podocalyxin expression in *GNE*-knockout cells was detectable only after increasing the exposure time. This, in turn, could be interpreted as further evidence of an actual effect on podocalyxin expression following *GNE*-knockout – especially given that the antibody was able to detect podocalyxin from sialidase-treated cells just as effectively as that from wild-type cells.

Whether hyposialylation also affects adhesion to a fibronectin-collagen IV substrate was investigated using a real time cell adhesion assay (Fig. 3d). We were able to demonstrate that adhesion in the *GNE*-knockout cells was significantly reduced by 44% (*p*-value 6.04E-03). In the cells treated with sialidase, we only observed a tendency towards lower adhesion abilities (*p*-value 6.82E-02). This is consistent with studies that have already demonstrated that the sialylation status of integrin’s – such as integrin beta-1 (also investigated here)^4,5^ or integrin alpha-4^6^– is of crucial importance for their interaction with, or adhesion to, ligands or substrates such as collagens. In these studies, higher sialylation correlated with stronger adhesion, which conversely implies that lower sialylation should correlate with weaker adhesion. This was clearly demonstrated in our *GNE*-knockout cells, whereby we attribute this point to reduced sialylation of integrin’s, rather than to reduced sialylation of podocalyxin, which, in principle, should not be found at the basolateral membrane anyway^39^.

### Determination to the recurrence time in western blot of normosialylated podocalyxin and integrin beta-1

We then wanted to investigate how long it takes after discontinuation of sialidase-treatment until normosialylated podocalyxin and integrin beta-1 is detectable and predominates again in the Western blot, or in other words, how long the cell needs to counteract the hyposialylation of these two proteins. For this purpose, we treated wild-type THBMECs with sialidase, harvested them at different time points, and examined the band patterns of both proteins by Western blot.

**Fig. 4:**
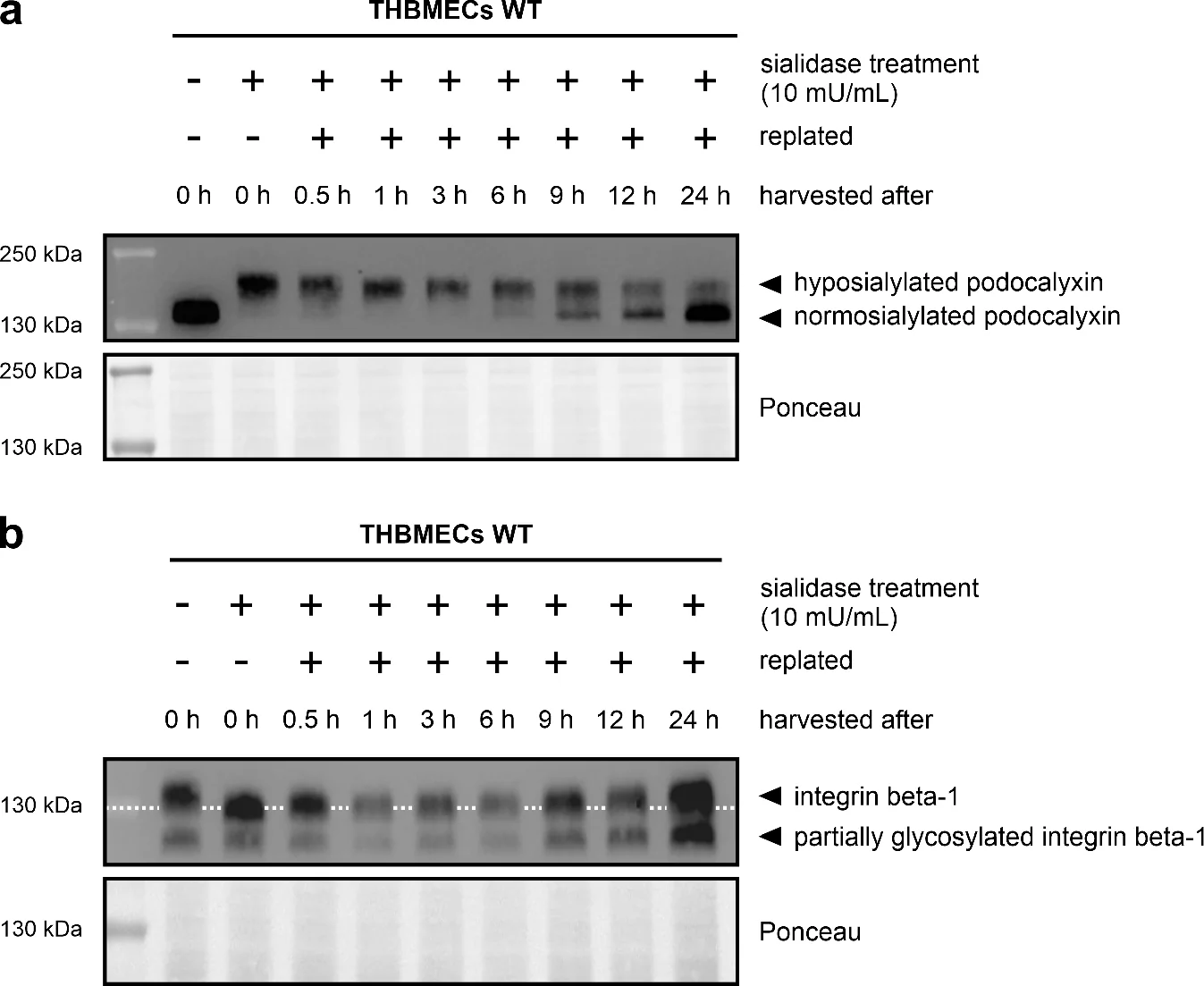
Determination of the recurrence time of sialylated proteins after treatment of wild-type THBMECs with sialidase. Wild-type THBMECs were treated with 10 mU/mL sialidase, replated, and harvested at different time points. A representative Western blot of podocalyxin with the corresponding Ponceau stain is shown in (**a**); the corresponding blots for integrin beta-1 are shown in (**b**). All Western blots resulted from three independent cell culture lyses that were investigated individually (n = 3). Furthermore, all Western blots were cropped. The white dashed line in (**b**) was added retrospectively to improve the visibility of a potential band shift. The original blots can also be found in the Supplementary Dataset File. Information concerning the antibody used and its concentration can be found in Table 1 in the Methods section.

In the blots, it was observed that – as previously demonstrated (Fig. 3a) – the electrophoretic mobility of podocalyxin was reduced after sialidase-treatment. This state changed only slightly during the first three hours (Fig. 4a). However, after six to nine hours, the sample slightly began to display two bands in the electric field and the following Western blot, indicating the reappearance of normosialylated podocalyxin. By the 24-hour mark, the fraction with lower electrophoretic mobility – representing hyposialylated podocalyxin – had almost completely disappeared.

For integrin beta-1, we observed, again as previously demonstrated, an increase in the electrophoretic mobility of the mature integrin following sialidase-treatment (Fig. 4b). At the time point thirty minutes after sialidase-treatment, the electrophoretic mobility of the mature integrin band shifted slightly, indicating now a lower electrophoretic mobility, but did not return to the level observed in untreated cells. However, this effect was not lasting, as the following time point showed. The “overshoot effect” 30 min after treatment and replating could have been caused by a rapid induction of sialylation of the partially glycosylated precursor protein – to mitigate cellular damage from a lack of sialylation. However, since we did not observe a drastic change in the signal intensity of the partially glycosylated integrin beta-1 between these two time points, it is quite unlikely that this is responsible for the main effect. Another explanation for this behavior could be that this fraction with lower electrophoretic mobility was based on recycled integrin beta-1; for instance in a clear-cell carcinoma cell line, 70% of the internalized integrin beta-1 returned to the cell membrane within 15 min^40^. Furthermore, Tringali et al. demonstrated that by silencing the naturally occurring sialidase Neu3 they could shift the fate of internalized integrin beta-1 proteins from recycling to lysosomal degradation^40^. In our case, however, this simply means that the administration of sialidase perhaps shifts the balance even more strongly towards recycling, at least initially. Nevertheless, the recovered integrin beta-1 should be fully glycosylated and thus also sialylated; why, then, does the electrophoretic mobility differ from that of the untreated sample, or why do we not observe two clear distinguishable bands? The difference in electrophoretic mobility between the two states - hypo- and normosialylated - may not be large enough resulting in a mixture of both states - that is, the higher the proportion of sialylated integrin beta-1, the lower the electrophoretic mobility.

Nine hours after replating, the electrophoretic mobility began to decrease slightly at each following time point, until it reached the level of the untreated cells after 24 h. As previously described, this can presumably be attributed to an increasing proportion of normosialylated integrin beta-1. Further, the timing of the reappearance is consistent with a study on WI-38 fibroblasts in which a half-life of approximately 10 h was determined for this protein^25^.

In summary, it can be stated that normosialylated podocalyxin was visible again in the Western blot after six hours and normosialylated integrin beta-1 one time point later - that is, at 9 h. Furthermore, thirty minutes after sialidase-treatment, the electrophoretic mobility of integrin beta-1 shifted back towards the normosialylated state for the first time. However, this was not sustained and can be seen as an indication that a comparatively rapid recycling pathway exists for integrin beta-1.

Subsequently, the hypothesis was investigated as to whether the change in the electrophoretic mobility of integrin beta-1 after nine hours was indeed - as suspected - attributable to the presence of newly synthesized and fully glycosylated proteins, or whether it resulted from recycling - but in this case, a comparatively long-lasting one. Furthermore, it should be investigated whether podocalyxin, which accounted for the band of normosialylated podocalyxin from the 6-hour mark onwards - originated from recycled or newly synthesized protein.

**Fig. 5:**
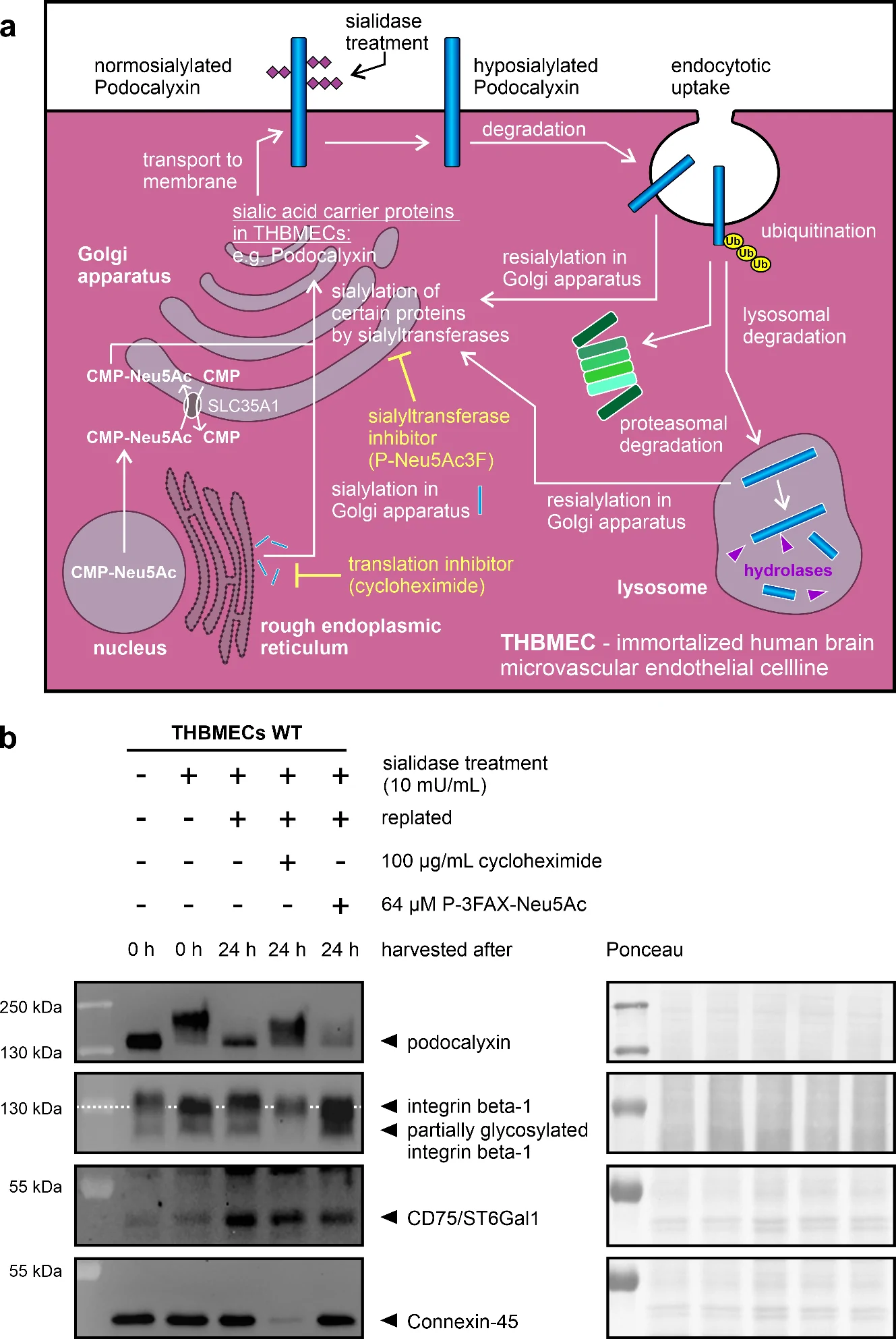
Determination of the origin of normosialylated podocalyxin and integrin beta-1 after discontinuation of sialidase-treatment. (**a**) shows a scheme with possible degradation pathways of hyposialylated proteins using podocalyxin as an example. Additionally, possible sialylation pathways and their influence by inhibitors are shown. To investigate which of these pathways is responsible for the reappearance of the normosialylated protein bands, wild-type cells were treated with 10 mU/mL sialidase for 30 min, replated, and then treated with either 100 µg/mL of the translation inhibitor cycloheximide or 64 µM of the sialyltransferase inhibitor P-3FAX-Neu5Ac. (**b**) shows a representative Western blot of podocalyxin, integrin beta-1, ST6Gal1, and connexin-45, along with the corresponding Ponceau staining. The Western blot experiments on podocalyxin, integrin beta-1, and connexin-45 were performed three times (n = 3), the experiments on ST6Gal1 twice (n = 2). Furthermore, all Western blots were cropped. The white dashed line in the Western blot for integrin beta-1 was added retrospectively to improve the visibility of a potential band shift. The original blots can also be found in the Supplementary Dataset File. Information concerning the antibodies used and their concentration can be found in Table 1 in the Methods section.

In principle, hyposialylated podocalyxin or proteins in general can undergo further processing in various ways (see scheme in Fig. 5a). They could be ubiquitinated, taken up, and subsequently degraded via the proteasomal or lysosomal pathway or they could be resialylated in the Golgi apparatus. The possibility of podocalyxin ubiquitination has been already demonstrated in a previous study^41^.

To investigate whether recycling or *de novo* synthesis predominates, wild-type THBMECs were treated with sialidase, replated, and subsequently treated with either the translation inhibitor cycloheximide or the sialyltransferase inhibitor P-3FAX-Neu5Ac. After 24 h, cells were harvested, and podocalyxin and integrin beta-1 expression was analyzed by Western blot.

As in previous experiments, we again observed that sialidase-treatment resulted in reduced electrophoretic mobility of podocalyxin and increased electrophoretic mobility of integrin beta-1 (Fig. 5b). This was completely reversed for both proteins 24 h after replating. The podocalyxin band from cycloheximide-treated cells still showed an upward shift, but it also smeared down to the normosialylated podocalyxin level. Following translation inhibition, integrin beta-1 exhibited the same electrophoretic mobility as it did immediately after sialidase-treatment. Interestingly, the band representing the partially glycosylated integrin beta-1 disappeared completely under these conditions.

The addition of cycloheximide is intended to prevent the *de novo* synthesis of proteins. Therefore, the location of the protein band after cycloheximide treatment can be used to determine the origin of the normosialylated protein after sialidase-treatment and replating. If the band in the Western blot appears at the level as that of the sialidase-treated cells, this can be interpreted as an indication that newly synthesized protein is responsible for the reappearance of the normosialylated protein band. Conversely, a band at the level of untreated cells can be interpreted as a sign for recycling. However, since we found the podocalyxin band after cycloheximide treatment of the cells mainly at the level of sialidase-treated cells, we conclude that *de novo* synthesis and not recycling is the cause for the reappearance of the normosialylated podocalyxin band in the Western blot. The same applies to integrin beta-1; furthermore, the disappearance of the partially glycosylated form in the Western blot indicates that this protein form most probably underwent further glycosylation and that no newly synthesized integrin beta-1 could replenish this protein pool, as translation had been successfully blocked.

Subsequently, we also verified that the cycloheximide concentration used was not lethal to the cells, yet high enough to efficiently block translation. We examined lethality by staining cells with trypan blue and found no significant differences between cycloheximide-treated and untreated cells (*p*-value 5.98E-02; Significance was determined using a two-sample *t*-test assuming different variances (one-sided). Raw data is available in the Supplementary Dataset File). In addition to the previously mentioned observation regarding the absence of the partially glycosylated integrin beta-1 band in the Western blot, the efficient inhibition of translation was also investigated by examining the expression of a protein with a short half-life. For this purpose, connexin-45 was chosen, which had a half-life of 4.2 h in HeLa cells transfected with mouse connexin-45^42^. In the Western blot, we observed a very strong decrease in the intensity of the connexin-45 band after treatment with cycloheximide (Fig. 5b), which we considered as confirmation that the concentration used was sufficient.

Furthermore, it must be ruled out that the persistence of the protein bands in the Western blot at the level of the sialidase-treated cells is only due to the fact that the involved sialyltransferases have such a short half-life that they are simply no longer present in sufficient quantities after cycloheximide treatment. We verified this by examining beta-galactoside alpha-2,6-sialyltransferase 1 (ST6Gal1) expression by Western blot in cycloheximide-treated and untreated cells (Fig. 5b). This sialyltransferase was chosen because, at least in mice, podocalyxin can exhibit glycan structures with sialic acids in α2,6-linkages^43^. Interestingly, the intensity of the ST6Gal1 band in the Western blot appears to increase after sialidase-treatment and replating compared to the untreated wild-type sample. Furthermore, sufficient protein appears to remain present after cycloheximide-treatment. This demonstrated that the persistence of the protein bands was presumably not attributable to the absence of sialyltransferases, although we examined only one as an example.

The next condition analyzed by Western blot was the addition of a sialyltransferase inhibitior. This should prevent any type of sialylation – whether on *de novo* synthesized or recycled proteins. In THBMECs treated with the sialyltransferase inhibitor, only a faint, smeared podocalyxin band was detected, with a tendency towards the normosialylated podocalyxin level. The integrin beta-1 band again exhibited the same electrophoretic mobility as immediately after sialidase-treatment and after translation inhibition. For integrin beta-1, it fits perfectly with the phenotype expected following sialyltransferase inhibition. The behavior of podocalyxin is not immediately fully comprehensible and requires further explanation. The reappearance or maybe better recognizability of the normosialylated podocalyxin band may possibly be explained by continuous degradation of hyposialylated podocalyxin, while the normosialylated podocalyxin, which might have escaped sialidase-treatment, became clearly visible again in the blot. However, this explanation for podocalyxin raised the question of why the band in the cycloheximide-treated cells does not appear equally faint. In a controlled system of *Xenopus* eggs with varying cytoplasmic protein concentrations, it was demonstrated that these concentrations influence translation and degradation rates. Specifically, when the cytoplasmic concentration drops below the normal physiological level, translation increases while degradation decreases^44^. Therefore, it is plausible to assume that the degradation rate in the P-3FAX-Neu5Ac cells is correspondingly higher, than in the cycloheximide-treated cells – which should lead to the degradation of hyposialylated podocalyxin and the fading of the band in the Western blot, as indeed it does under the given conditions.

Overall, we conclude that both *GNE*-knockout and sialidase-treatment of THBMECs resulted in a sialic acid deficiency, which could be rescued in the knockout by supplementation with ManNAc or Neu5Ac. Furthermore, the cells exhibited reduced membrane permeability, which could be interpreted as an indication of a tighter barrier, as well as a reduced ability of the cells to adhere to a fibronectin-collagen IV substrate. Six hours after sialidase-treatment, normosialylated podocalyxin was again detected; this was largely attributable to newly synthesized, rather than recycled, podocalyxin. Normosialylated integrin beta-1 reappeared in the Western blot after the nine-hour time point; this is likewise attributable to newly synthesized integrin. Furthermore, we found evidence of integrin recycling, though only as a rapid response - specifically, thirty minutes after sialidase-treatment.

## Methods

### Generation of a THBMEC GNE-knockout cell line

The transfected human brain microvascular endothelial cells (THBMECs) were kindly provided by the Danker lab (originally from^12^). THBMEC *GNE*-knockout cells were generated by a co-transfection of a CRISPR/Cas9-KO (sc-406100) and a HDR plasmid (sc-406100-HDR), both obtained from Santa Cruz Biotechnology (Dallas, TX, USA). Afterwards, the cells were sorted by flow cytometry. Single cell clones were analyzed for knockout success using Western blotting and two different GNE-antibodies - one of them recognizing an epitope at the C-terminus and one at the N-terminus.

### Cell culture of THBMEC wild-type and GNE-knockout

Both cell lines were cultured in DMEM/F12 supplemented with 10% FCS, 1% penicillin/streptomycin, and 1% L-glutamine in a humidified atmosphere at 37 °C with 5% CO_2_. In addition, they were checked for mycoplasma contamination after each thawing. For the experiments, they were seeded at a density of one million cells per 10 cm (Ø) cell culture dish. After 48 h, the medium was changed - additions, e.g. 5 mM ManNAc (solved in 1xPBS; New Zealand Pharmaceuticals, Palmerston North, New Zealand) or 5 mM Neu5Ac (solved in 1xPBS; 39596039; Molekula Group; Munich, Germany), to the new medium can be made here - and after another day, the cells were harvested in RIPA buffer or directly in SDS-sample buffer (ManNAc and Neu5Ac-supplementation experiment).

### SDS-PAGE and western blot

Total protein content of samples harvested in RIPA buffer was determined using the Pierce™ BCA protein assay kit (A65453; Thermo Fisher Scientific; Waltham, MA, USA). Then, 30 µg of total protein was added to the SDS-sample buffer and heated at 95 °C for 5 min. For SDS-PAGE, this was then loaded onto a 10% gel.

Samples harvested directly into sample buffer were heated at 95 °C for 5 min and then 8 µl were loaded onto a 10% gel.

The proteins were then blotted onto a nitrocellulose membrane (0.45 μm pore size; Amershan™ Protran™ 0.45 NC, roll) and stained with Ponceau S as a loading control. The membrane was then blocked for one hour with 5% milk in Tris-buffered saline and 0.1% Tween 20 (TBS-T). The blocking solution was then removed, the primary antibody solution was added (see Table 1), and the blot was incubated overnight at 4 °C with continuous shaking. After three 5-minute wash cycles with TBS-T, the secondary antibody solution was incubated for one hour at room temperature with continuous shaking. The washing procedure was repeated, with the difference that each washing step now lasted 10 min, and then the blot was investigated using the ChemiDoc MP imaging system (Bio-Rad Laboratories; Hercules, CA, USA) after addition of the HRP substrate (WBLUF0500; Millipore/Merck KGaA; Darmstadt, Germany).

For lectin staining, we adapted the protocol of Lerrer and Gilboa-Garber for our Alexa647-labeled PNA lectin^45^. Following blotting, the membrane was first blocked overnight at 4 °C with 3% BSA in PBS and 0.05% Tween-20. Subsequently, the blocking solution was removed, the lectin-solution was added (see Table 1), and the blot was incubated for two hours at room temperature with continuous shaking. After four 10-minute wash cycles with PBS and 0.1% Tween-20 (PBS-T), the blot was investigated using the ChemiDoc MP imaging system and the corresponding Alexa647 channel.

**Table 1:** List of antibodies used in Western Blot; RRID: Research Resource Identifier; TBS-T: Tris-buffered saline supplemented with 0.1% Tween 20; HRP: horse radish peroxidase.

Name	Target protein/ modification	Clonality and species	Dilution	Company and ordering number	RRID
Primary Antibodies					
CMAS antibody (E-8)	CMAS (UniProt ID: Q8NFW8)	Monoclonal, mouse	1:1000 in TBS-T	Santa Cruz Biotechnology, sc-398296	-
Connexin 45 antibody (G-7)	Gap junction gamma-1 protein (UniProt ID: P36383)	Monoclonal, mouse	1:1000 in TBS-T	Santa Cruz Biotechnology, sc-374354	AB_10988777
GlcNAc kinase antibody (G-5)	NAGK (UniProt ID: Q9UJ70)	Monoclonal, mouse	1:1000 in TBS-T	Santa Cruz Biotechnology, sc-390499	-
GLCNE antibody (H-10)	GNE; N-terminal (UniProt ID: Q9Y223)	Monoclonal, mouse	1:1000 in TBS-T	Santa Cruz Biotechnology, sc-376057	AB_10988883
anti-GNE antibody [EPR15059]	GNE; C-terminal (UniProt ID: Q9Y223)	Monoclonal, rabbit	1:1000 in TBS-T	abcam, ab189927	-
Integrin beta-1 antibody (D2E5)	Integrin beta-1 (UniProt ID: P05556)	Monoclonal, rabbit	1:1000 in 5% BSA + TBS-T	Cell Signaling, 9699	AB_11178800
NANP antibody (D8)	NANP (UniProt ID: Q8TBE9)	Monoclonal, mouse	1:1000 in TBS-T	Santa Cruz Biotechnology, sc-374637	AB_10989982
NANS antibody (B6)	NANS (UniProt ID: Q9NR45)	Monoclonal, mouse	1:1000 in TBS-T	Santa Cruz Biotechnology, sc-374133	AB_10917934
Podocalyxin-like 1 antibody (3D3)	Podocalyxin (UniProt ID: O00592)	Monoclonal, mouse	1:1000 in TBS-T	Santa Cruz Biotechnology, sc-23904	AB_2166006
Anti-CD75 antibody [EPR22054-238]	ST6GAL1 (UniProt ID: P15907)	Monoclonal, rabbit	1:1000 in TBS-T	abcam, ab236461	-
Lectin					
PNA from *Arachis hypogaea* (peanut), Alexa Fluor 647 Conjugate	Terminal galactose	-	1 µg/ml in 3% BSA + PBS-T	Thermo Fisher Scientific, L32460	-
Secondary Antibodies					
Anti-mouse IgG, HRP-linked antibody	Mouse IgG	Polyclonal, horse	1:5000 in TBS-T	Cell Signaling, 7076	AB_330924
Anti-rabbit IgG-HRP	Rabbit IgG	Monoclonal, mouse	1:5000 in TBS-T	Santa Cruz Biotechnology, sc-2357	AB_628497

### Sialidase-treatment

THBMECs were seeded at a density of one million cells per 10 cm (Ø) cell culture dish. After 48 h, the medium was removed and the dishes were washed once with PBS. Afterwards, the cells were harvested in PBS using a cell scraper and centrifuged at 1500 rpm for three minutes. The supernatant was discarded, the cell pellet was resuspended in medium and the number of cells in the suspension was determined. Per 1.5 mL reaction tube, 2.5 million cells in 1 mL medium and 10 mU/mL sialidase from *Arthrobacter ureafaciens* (10269611001; Roche/Merck KGaA; Darmstadt, Germany) were added and each tube was incubated at 37 °C for 30 min. The tube was then centrifuged at 1500 rpm for three minutes, the supernatant was discarded, and the pellet was resuspended in PBS and centrifuged again.

The cells were then plated and the medium was changed after one day in culture. After another day, the cells were harvested in RIPA buffer supplemented with PMSF, sodium orthovanadate and protease inhibitor cocktail (sc-24948; Santa Cruz Biotechnology; Dallas, TX, USA) and then analyzed by Western blot. For the band shift rescue experiments, cells were plated and harvested in 1xSDS-sample buffer after 30 min, 1 h, 3 h, 6 h, 9 h, 12 h or 24 h; no additional medium exchange was carried out. For the podocalyxin recycling experiment, cells were plated in medium containing 100 µg/mL cycloheximide (J66004.XF; Thermo Fisher Scientific; Waltham, MA, USA) or in medium containing 64 µM P-3FAX-Neu5Ac (SV4037; Synvenio; Nijmegen, The Netherlands) and harvested in 1xSDS-sample buffer after 24 h. Cell viability was tested in the podocalyxin recycling experiment using trypan blue staining.

### Periodate-resorcinol assay for the determination of sialic acid content

Our assay was based on the protocol of Dammen-Brower et al. with minor modifications^46^. Cells were treated as previously described – see sialidase-treatment of THBMEC wild-type cells and ManNAc and Neu5Ac supplementation of THBMEC *GNE*-KO cells. The cells were then harvested, washed three times with 1xPBS, counted, two replicates of each condition containing one million cells each were prepared, centrifuged and resuspended in 250 µL of 1xPBS. The cells were then disrupted by freeze-thaw lysis performed three times. To create a sialic acid standard curve, samples with different sialic acid concentrations were prepared. Subsequently, 0.5 µL of 0.4 M periodic acid was added to each sample, including the standard curve, and mixed thoroughly. One replicate of each condition and the standard curve samples were then incubated on ice for 90 min. The other replicate was incubated for the same time at 37 °C. Afterwards, 500 µL of a solution consisting of 0.6% resorcinol, 0.25 mM CuSO_4_, 36% water, and 44% concentrated HCl were added to each sample, mixed thoroughly and heated to 100 °C for 15 min (be careful during this step; wear protective googles and use special, safety-sealed reaction tubes). All samples were allowed to cool to room temperature, 500 µL of *tert*-butyl alcohol were added, mixed thoroughly, centrifuged, equal volumes of the supernatant of each sample were pipetted into a 96-well plate and the absorbance at 630 nm was determined. This value was subsequently correlated with the simultaneously generated standard curve to determine the sialic acid concentration of the samples.

### Measurement of the transepithelial electrical resistance

Millicell 12-well hanging cell culture inserts (pore size 0.4 μm; PTHT12H48; Merck Millipore; Burlington; MA; USA) were coated with 10 µg/mL human collagen IV (C5533; Sigma Aldrich; St. Louis, MO; USA) and 10 µg/mL human fibronectin (F0895; Sigma Aldrich; St. Louis, MO; USA). Afterwards, the cells were seeded at a density of 100k per cell culture insert and treated with the indicated substances. Every two days the medium was changed and the treatment refreshed. After 14 days, the transepithelial electrical resistance was determined using the Millicell ERS 3.0 digital voltohmmeter (MERS03000; Merck Millipore; Burlington, MA; USA).

### Real time cell adhesion assay

E-Plates (Agilent) were coated with 10 µg/mL human collagen IV and 10 µg/mL human fibronectin and washed twice with PBS. Cells were treated with sialidase prior to seeding as previously described. Additionally, *GNE*-knockout cells were treated with 5 mM ManNAc 48 h before adhesion measurements. The pretreated - sialidase or ManNAc - and the untreated cells were seeded at a density of 10k per well and measured using the xCELLigence RTCA instrument (Agilent Technologies; Santa Clara, CA; USA).

### Statistics and reproducibility

For statistical evaluation, we used a one-sided two-sample *t*-test with assumed different variances. The exact *p*-values, the *t*-statistic, and degrees of freedom, along with the mean, standard deviation, and variance can be found in the Supplementary Dataset File. For the representation of significances we used the following depiction: 1.00E-02 < *p* ≤ 5.00E-02: *, 5.00E-03 < *p* ≤ 1.00E-02: **, and *p* ≤ 5.00E-03: ***. All experiments were performed triplicate – based on three independent cell lyses/cell culture dishes, except for the ST6Gal1/CD75 blot, which was performed only twice. For TEER and RTCA measurements: For each approach, three technical replicates were prepared and averaged – giving the value for one biological replicate. Each approach was performed in triplicate.

## Supplementary Information

Below is the link to the electronic supplementary material.

Supplementary Material 1

## Data Availability

All data generated or analyzed during this study are included in this published article and its Supplementary Dataset File.

## References

[1] Su, D., Zhao, H. & Xia, H. Glycosylation-modified erythropoietin with improved half-life and biological activity. *Int. J. Hematol.* **91**, 238–244 (2010). 20131103 10.1007/s12185-010-0496-x

[2] Byrne, C. et al. Enhanced α2-3-linked sialylation determines the extended half-life of CHO-rVWF. *Blood* **145**, 2768–2773 (2025). 40101144 10.1182/blood.2024027038 PMC12824655

[3] Morell, A. G., Gregoriadis, G., Scheinberg, I. H., Hickman, J. & Ashwell, G. The role of sialic acid in determining the survival of glycoproteins in the circulation. *J. Biol. Chem.* **246**, 1461–1467 (1971). 5545089

[4] Seales, E. C. et al. Hypersialylation of beta1 integrins, observed in colon adenocarcinoma, may contribute to cancer progression by up-regulating cell motility. *Cancer Res.* **65**, 4645–4652 (2005). 15930282 10.1158/0008-5472.CAN-04-3117

[5] Lee, M., Lee, H. J., Seo, W. D., Park, K. H. & Lee, Y. S. Sialylation of integrin beta1 is involved in radiation-induced adhesion and migration in human colon cancer cells. *Int. J. Radiat. Oncol. Biol. Phys.* **76**, 1528–1536 (2010). 20338479 10.1016/j.ijrobp.2009.11.022

[6] Natoni, A. et al. Sialyltransferase inhibition leads to inhibition of tumor cell interactions with E-selectin, VCAM1, and MADCAM1, and improves survival in a human multiple myeloma mouse model. *Haematologica* **105**, 457–467 (2020). 31101754 10.3324/haematol.2018.212266 PMC7012485

[7] Yogeeswaran, G. & Salk, P. L. Metastatic potential is positively correlated with cell surface sialylation of cultured murine tumor cell lines. *Science* **212**, 1514–1516 (1981). 7233237 10.1126/science.7233237

[8] Yang, L. et al. Aberrant sialic acid metabolism promotes metabolic reprogramming and metastasis in breast cancer. *Front. Oncol.* **15**, 1698087. 10.3389/fonc.2025.1698087 (2025). 41333208 10.3389/fonc.2025.1698087 PMC12665555

[9] Baycin-Hizal, D. et al. Physiologic and pathophysiologic consequences of altered sialylation and glycosylation on ion channel function. *Biochem. Biophys. Res. Commun.* **453**, 243–253 (2014). 24971539 10.1016/j.bbrc.2014.06.067 PMC4544737

[10] Muchmore, E. A., Diaz, S. & Varki, A. A structural difference between the cell surfaces of humans and the great apes. *Am. J. Phys. Anthropol.* **107**, 187–198 (1998). 9786333 10.1002/(SICI)1096-8644(199810)107:2<187::AID-AJPA5>3.0.CO;2-S

[11] Varki, A. Colloquium paper: uniquely human evolution of sialic acid genetics and biology. *Proc. Natl. Acad. Sci. U S A*. **107** (Suppl 2), 8939–8946 (2010). 20445087 10.1073/pnas.0914634107 PMC3024026

[12] Stins, M. F., Badger, J. & Sik Kim, K. Bacterial invasion and transcytosis in transfected human brain microvascular endothelial cells. *Microb. Pathog*. **30**, 19–28 (2001). 11162182 10.1006/mpat.2000.0406

[13] Labus, J., Häckel, S., Lucka, L. & Danker, K. Interleukin-1β induces an inflammatory response and the breakdown of the endothelial cell layer in an improved human THBMEC-based in vitro blood-brain barrier model. *J. Neurosci. Methods*. **228**, 35–45 (2014). 24631939 10.1016/j.jneumeth.2014.03.002

[14] Weber, V., Bork, K., Horstkorte, R. & Olzscha, H. Analyzing the Permeability of the Blood-Brain Barrier by Microbial Traversal through Microvascular Endothelial Cells. *J. Vis. Exp.* **14** (156). 10.3791/60692 (2020). 10.3791/60692 32116297

[15] Kerjaschki, D., Sharkey, D. J. & Farquhar, M. G. Identification and characterization of podocalyxin–the major sialoprotein of the renal glomerular epithelial cell. *J. Cell. Biol.* **98**, 1591–1596 (1984). 6371025 10.1083/jcb.98.4.1591 PMC2113206

[16] Kitano, M. et al. Rab11-mediated post-Golgi transport of the sialyltransferase ST3GAL4 suggests a new mechanism for regulating glycosylation. *J. Biol. Chem.* **296**, 100354. 10.1016/j.jbc.2021.100354 (2021). 33524390 10.1016/j.jbc.2021.100354 PMC7949161

[17] Cait, J. et al. Podocalyxin is required for maintaining blood-brain barrier function during acute inflammation. *Proc. Natl. Acad. Sci. U S A*. **116**, 4518–4527 (2019). 30787191 10.1073/pnas.1814766116 PMC6410846

[18] Keppler, O. T. et al. UDP-GlcNAc 2-epimerase: a regulator of cell surface sialylation. *Science* **284**, 1372–1376 (1999). 10334995 10.1126/science.284.5418.1372

[19] Peters, E., Selke, P., Bork, K., Horstkorte, R. & Gesper, A. Evaluation of N-Acetylmannosamine Administration to Restore Sialylation in GNE-Deficient Human Embryonal Kidney Cells. *Front. Biosci. (Landmark Ed.* **28**, 300. 10.31083/j.fbl2811300 (2023). 38062838 10.31083/j.fbl2811300

[20] Weidemann, W. et al. Lessons from GNE-deficient embryonic stem cells: sialic acid biosynthesis is involved in proliferation and gene expression. *Glycobiology* **20**, 107–117 (2010). 19797319 10.1093/glycob/cwp153

[21] Kershaw, D. B. et al. Molecular cloning and characterization of human podocalyxin-like protein. Orthologous relationship to rabbit PCLP1 and rat podocalyxin. *J. Biol. Chem.* **272**, 15708–15714 (1997). 9188463 10.1074/jbc.272.25.15708

[22] Tamkun, J. W. et al. Structure of integrin, a glycoprotein involved in the transmembrane linkage between fibronectin and actin. *Cell* **46**, 271–282 (1986). 3487386 10.1016/0092-8674(86)90744-0

[23] Dekan, G., Gabel, C. & Farquhar, M. G. Sulfate contributes to the negative charge of podocalyxin, the major sialoglycoprotein of the glomerular filtration slits. *Proc. Natl. Acad. Sci. U S A*. **88**, 5398–5402 (1991). 2052617 10.1073/pnas.88.12.5398 PMC51880

[24] Takeda, T., Go, W. Y., Orlando, R. A. & Farquhar, M. G. Expression of podocalyxin inhibits cell-cell adhesion and modifies junctional properties in Madin-Darby canine kidney cells. *Mol. Biol. Cell.* **11**, 3219–3232 (2000). 10982412 10.1091/mbc.11.9.3219 PMC14987

[25] Heino, J., Ignotz, R. A., Hemler, M. E., Crouse, C. & Massagué, J. Regulation of cell adhesion receptors by transforming growth factor-beta. Concomitant regulation of integrins that share a common beta 1 subunit. *J. Biol. Chem.* **264**, 380–388 (1989). 2491849

[26] Gahmberg, C. G. & Andersson, L. C. Role of sialic acid in the mobility of membrane proteins containing O-linked oligosaccharides on polyacrylamide gel electrophoresis in sodium dodecyl sulfate. *Eur. J. Biochem.* **122**, 581–586 (1982). 7060593 10.1111/j.1432-1033.1982.tb06478.x

[27] Lotan, R., Skutelsky, E., Danon, D. & Sharon, N. The purification, composition, and specificity of the anti-T lectin from peanut (Arachis hypogaea). *J. Biol. Chem.* **250**, 8518–8523 (1975). 811657

[28] Galeano, B. et al. Mutation in the key enzyme of sialic acid biosynthesis causes severe glomerular proteinuria and is rescued by N-acetylmannosamine. *J. Clin. Invest.* **117**, 1585–1594 (2007). 17549255 10.1172/JCI30954 PMC1878529

[29] Ito, M. et al. Glycoprotein hyposialylation gives rise to a nephrotic-like syndrome that is prevented by sialic acid administration in GNE V572L point-mutant mice. *PLoS One*. **7**, e29873. 10.1371/journal.pone.0029873 (2012). 22253810 10.1371/journal.pone.0029873 PMC3258264

[30] Gorenflos López, J. L. et al. Real-time monitoring of the sialic acid biosynthesis pathway by NMR. *Chem. Sci.* **14**, 3482–3492 (2023). 37006695 10.1039/d2sc06986e PMC10055903

[31] Allen, M. B. & Walker, D. G. Kinetic characterization of N-acetyl-D-glucosamine kinase from rat liver and kidney. *Biochem. J.* **185**, 577–582 (1980). 6248026 10.1042/bj1850577 PMC1161433

[32] Hinderlich, S., Berger, M., Keppler, O. T., Pawlita, M. & Reutter, W. Biosynthesis of N-acetylneuraminic acid in cells lacking UDP-N-acetylglucosamine 2-epimerase/N-acetylmannosamine kinase. *Biol. Chem.* **382**, 291–297 (2001). 11308027 10.1515/BC.2001.036

[33] Kakani, S. et al. The Gne M712T mouse as a model for human glomerulopathy. *Am. J. Pathol.* **180**, 1431–1440 (2012). 22322304 10.1016/j.ajpath.2011.12.023 PMC3349896

[34] Carrillo, N., Malicdan, M. C., Huizing, M. G. N. E. & Myopathy Etiology, Diagnosis, and Therapeutic Challenges. *Neurotherapeutics* **15**, 900–914 (2018). 30338442 10.1007/s13311-018-0671-y PMC6277305

[35] Stark, C. et al. BioGRID: a general repository for interaction datasets. *Nucleic Acids Res.* **34** (Database issue), D535–D539 (2006). 16381927 10.1093/nar/gkj109 PMC1347471

[36] Li, W., Maloney, R. E., Circu, M. L., Alexander, J. S. & Aw, T. Y. Acute carbonyl stress induces occludin glycation and brain microvascular endothelial barrier dysfunction: role for glutathione-dependent metabolism of methylglyoxal. *Free Radic Biol. Med.* **54**, 51–61 (2013). 23108103 10.1016/j.freeradbiomed.2012.10.552 PMC3742316

[37] Doyonnas, R. et al. Anuria, omphalocele, and perinatal lethality in mice lacking the CD34-related protein podocalyxin. *J. Exp. Med.* **194**, 13–27 (2001). 11435469 10.1084/jem.194.1.13 PMC2193439

[38] Tulsiani, D. R. & Carubelli, R. Studies on the soluble and lysosomal neuraminidases of rat liver. *J. Biol. Chem.* **245**, 1821–1827 (1970). 5438365

[39] Horvat, R., Hovorka, A., Dekan, G., Poczewski, H. & Kerjaschki, D. Endothelial cell membranes contain podocalyxin–the major sialoprotein of visceral glomerular epithelial cells. *J. Cell. Biol.* **102**, 484–491 (1986). 3511072 10.1083/jcb.102.2.484 PMC2114082

[40] Tringali, C. et al. The plasma membrane sialidase NEU3 regulates the malignancy of renal carcinoma cells by controlling β1 integrin internalization and recycling. *J. Biol. Chem.* **287**, 42835–42845 (2012). 23139422 10.1074/jbc.M112.407718 PMC3522280

[41] Yuan, Y. P. et al. The SGK3-triggered ubiquitin-proteasome degradation of podocalyxin (PC) and ezrin in podocytes was associated with the stability of the PC/ezrin complex. *Cell. Death Dis.* **9**, 1114. 10.1038/s41419-018-1161-1 (2018). 30385740 10.1038/s41419-018-1161-1 PMC6212497

[42] Hertlein, B., Butterweck, A., Haubrich, S., Willecke, K. & Traub, O. Phosphorylated carboxy terminal serine residues stabilize the mouse gap junction protein connexin45 against degradation. *J. Membr. Biol.* **162**, 247–257 (1998). 9543497 10.1007/s002329900362

[43] Weinhold, B. et al. Deficits in sialylation impair podocyte maturation. *J. Am. Soc. Nephrol.* **23**, 1319–1328 (2012). 22745475 10.1681/ASN.2011090947 PMC3402283

[44] Chen, Y., Huang, J. H., Phong, C. & Ferrell, J. E. Jr Viscosity-dependent control of protein synthesis and degradation. *Nat. Commun.* **15**, 2149. 10.1038/s41467-024-46447-w (2024). 38459041 10.1038/s41467-024-46447-w PMC10923802

[45] Lerrer, B. Gilboa-Garber, N. Differential staining of western blot of avian egg white glycoproteins using diverse lectins. *Electrophoresis* **23**, 8–14 (2002). 11824624 10.1002/1522-2683(200201)23:1<8::AID-ELPS8>3.0.CO;2-T

[46] Dammen-Brower, K., Tan, E., Almaraz, R. T., Du, J. & Yarema, K. J. Protocol Considerations for In Vitro Metabolic Glycoengineering of Non-Natural Glycans. *Curr. Protoc.* **3**, e822. 10.1002/cpz1.822 (2023). 37358193 10.1002/cpz1.822

